# Supplementary material for: Mesoporous nanoperforators as membranolytic agents via nano- and molecular-scale multi-patterning
Source: Nat Commun. 2024 Feb 29;15:1891. doi: 10.1038/s41467-024-46189-9 (PMC10904871; doi:10.1038/s41467-024-46189-9)
Supplement: Supplementary file 1 — Supplementary Information [file 41467_2024_46189_MOESM1_ESM.pdf]

Supplementary Information

Mesoporous Nanoperforators as Membranolytic Agents *via* Nano- and Molecular-scale Multi-patterning

Yannan Yang*, Shiwei Chen, Min Zhang*, Yiru Shi, Jiangqi Luo, Yiming Huang, Zhengying Gu, Wenli Hu, Ye Zhang, Xiao He* and Chengzhong Yu*

Force field parameters for molecules. The DPPC lipid, nanotube and water molecules were modelled using the MARTINI force field. The model is developed based on structural properties of molecules and partitioning free energies in different solvents and based on a four-to-one mapping of heavy atoms. Within this approach, the headgroup of a DPPC lipid molecule is composed of two types of coarse-grained beads corresponding to polar (Q) and nonpolar (N) beads. The tail group is composed of apolar (C) beads. MARTINI coarse-grained molecular structures of DPPC lipids used in the present study are schematically illustrated as below.

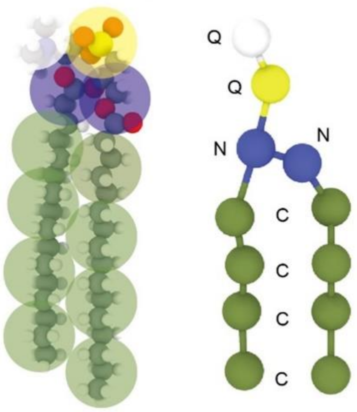

Schematic illustration of the coarse graining model of DPPC molecular in Martini force field, where white ball Q present charged particles, blue ball N present non-polar particle and green ball C present aploar particles.

The nanotube is composed of benzene bridged organosilica with a molecular scale ordering (schematically illustrated as below). In this case, only two kinds of beads are necessary to map the molecule: one for the [$(CH)_2$] in benzene groups (Small Molecules, Adv. Theory and Sims. 2022, 5, 2100391) and one for the [$O_{1/2}$-Si(C)-$O_{1/2}$] groups (Soft Matter, 2022, 18, 7887-7896). According to the standard mode (Nature, 2002, 416, 304-307.), there are 60 -Si- and 30 benzene at each lap. After coarse graining, there are 60 [$O_{1/2}$-Si(C)- $O_{1/2}$] groups and 60 benzene [$(CH)_2$] groups at each lap. The diameter and the length were set to 4.0 nm and 8.7 nm, respectively. The water particle has no charge and interacts with other particles through Lennard-Jones interactions. To simplify the simulation, we set the nanotube as a rigid structure. The quantum calculations were performed using restricted wB97XD with the 6-311+G* basis set.

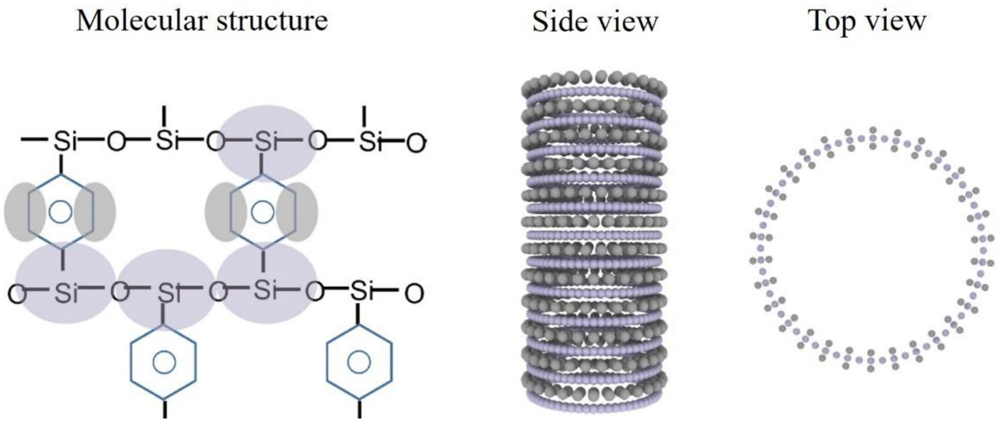

Schematic illustration of the coarse graining model of nanotube in Martini force field, where purple ball present [$O_{1/2}$-Si(C)-$O_{1/2}$] group and grey ball present $[CH]_2$ group.

System preparation and general course-grained molecular dynamics simulation.
(1) Equilibrium conformation of the system. The initial structures and topologies for nanotubes were prepared using the Moltemplate program. The dimensions of the system are 34 nm × 34 nm × 35 nm. The lower membrane is 10 nm away from the bottom of the box, while the upper membrane is 20 nm away from the top. The systems were consisted of 4608 DPPC lipids and 200900 water molecules. The length and diameter of the nanotubes are 8.7 nm and 4.0 nm, respectively. Typically, three types of rigid nanotube, namely, single, sector and cone forms, were placed 12 nm above the upper membrane, separately. To keep consistent with experiment, the diameter of nanotube was set to 4 nm. In all simulations, the temperature was kept constant at 310 K by the Nosé-Hoover algorithm. The system was relaxed using an NPT (the constant-temperature, constant-pressure) ensemble for 10 ns. Short-ranged electrostatic interactions are cut off at 0.9 nm, while van de Waals interactions are cut off at 1.2 nm. All simulations were performed by the LAMMPS package.
(2) Non-equilibrium molecular dynamics (NEMD) simulation of nanotube(s) through DPPC membranes. After obtaining the stable system, the NVT (the constant-temperature, constant-volume) ensemble was used in simulation. We conducted the NEMD simulations with an external force (0.01 kcal $mol^{-1}Å^{-1}$) since this allows gaining the phenomenon of membrane permeation within the limited simulation time. The timestep was set to 1 fs and the system was simulated for 1 ns. The pore areas as a function of distance between nanotube and bilayer membrane was calculated by python code. The radical distribution function was calculated by Visual Machine Design (VMD) software.

Molecular structure preparation and quantum chemistry calculation.
(1) Structure optimization. The molecular structure of DPPC lipid unit cell was built by Gauss View software. The molecules were optimized using the Gaussian 16

program with the wB97XD/6-311+G*. To simplify the system, all atoms but benzenes were removed in nanotube unit. Two benzenes were placed beside the DPPC lipid at a series of distance, namely, 2.5, 3.0, 3.5, 4.0, 4.5, 5.0, 6.0, 6.5 and 7.0 Å, respectively.

(2) **Binding energy calculation.** After obtaining the optimized molecular structure, single point energy of each molecule as well as the combined structure was calculated using ωB97XD/6-311+G* with the Gaussian16 program. The binding energy equals to the difference between the single point energy of combined structure and that of single molecule. Considering the lack of perfect structure of nanotube, we did not optimize the complex. In simplest terms, the benzene dimer is part of the nanotube, we ignore the Si and O connected with benzene, therefore, the possible contact structure for benzene dimer is side contact, while the optimized structure is top contact. To make the calculation more reliable, we optimize the benzene and DPPC molecular with the ωB97XD/6-311+G* basis set and calculate the binding energy of three type of benzene groups in three typical positions as shown in Supplementary Figure 21. To make the calculation more reliable, we calculate the binding energy of three type of benzene groups in three typical positions. The binding energy of parallel structure is lowest at all three positions. And the highest binding energy value (-6.5 kcal/mol) of parallel structure in three position is lower than the lowest binding energy value (-4.5 kcal/mol) of perpendicular and parallel-perpendicular structure. Such multiple position sampling calculation can help to guarantee the interesting result that keeping the planes of each benzene ring positioned parallelly to the axial direction of the phospholipid molecule could lead to minimum binding energy. Remarkably, ωB97XD/6-311+G* is a middle lever basis set and the error is about 0.4 kcal/mol (ref: Computational and Theoretical Chemistry, 2016, 1098: 1-12).

Statistics and reproducibility. All statistical analyses were performed using GraphPad Prism 8.0.2. Data were analysed using unpaired two-tailed Student's *t*-tests for the calculation of *P* values. The number of replicates performed is indicated in each figure legend, where applicable.

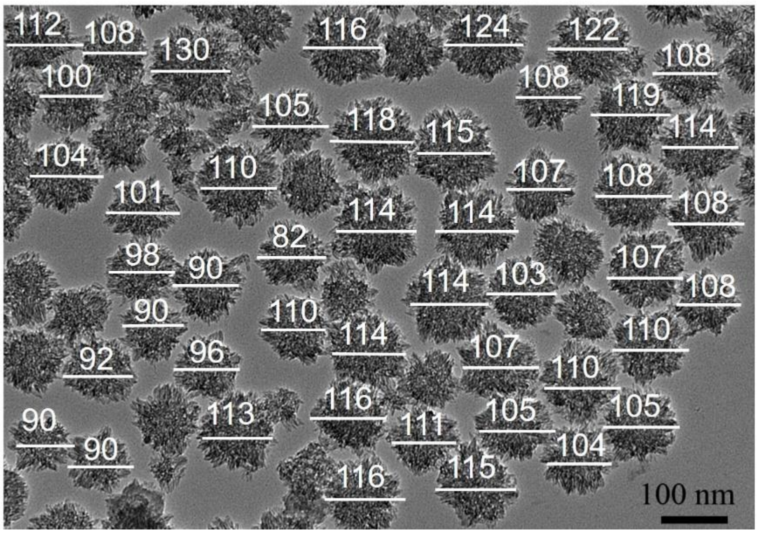

Supplementary Figure 1. Zoom-out TEM image of MLNPs. The diameters of 50 nanoparticles are labeled.

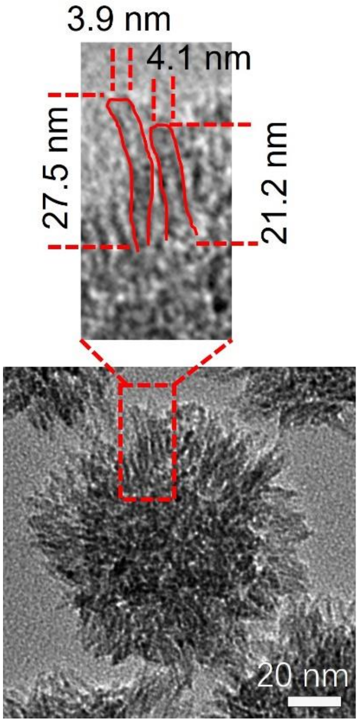

Supplementary Figure 2. TEM image for revealing the dimension of surface spikes of MLNPs.

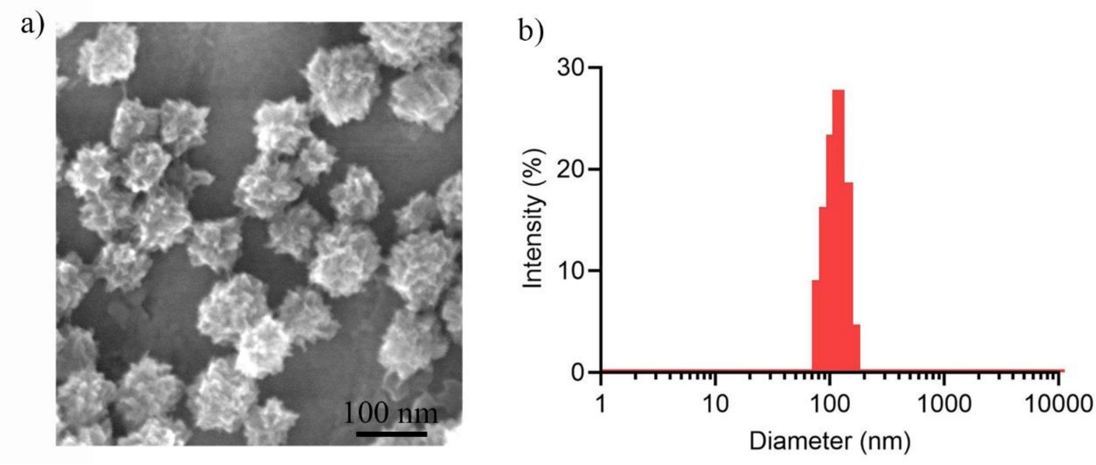

Supplementary Figure 3. a) SEM image and b) DLS analysis of MLNPs.

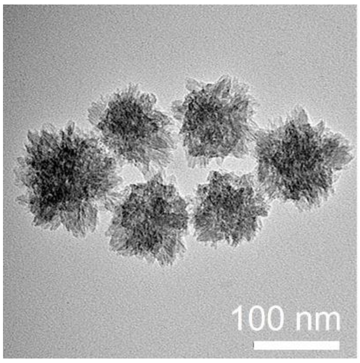

Supplementary Figure 4. The TEM image of MLNPs after immersing in PBS at 37°C for 7 days.

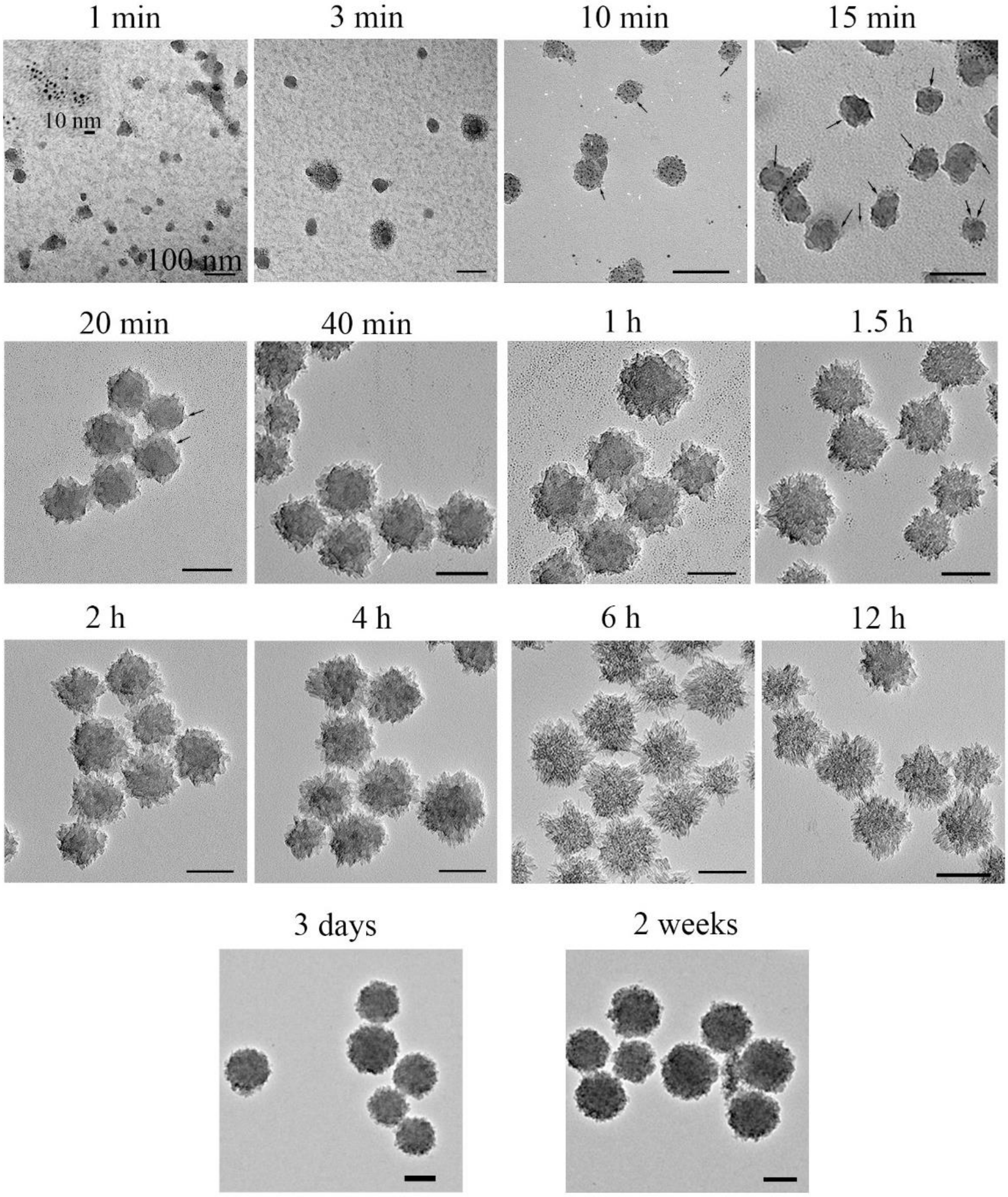

Supplementary Figure 5. Time-dependant TEM images of MLNPs. Inset: a high-magnification TEM image for primary organosilica particles. Black arrows indicate the hollow spheres formed by the assembly of CTAB micelles and organosilica oligomer. White arrows indicate the formation of hollow-structured surface spikes at 40 min. Except for the inset, all the scale bars are 100 nm.

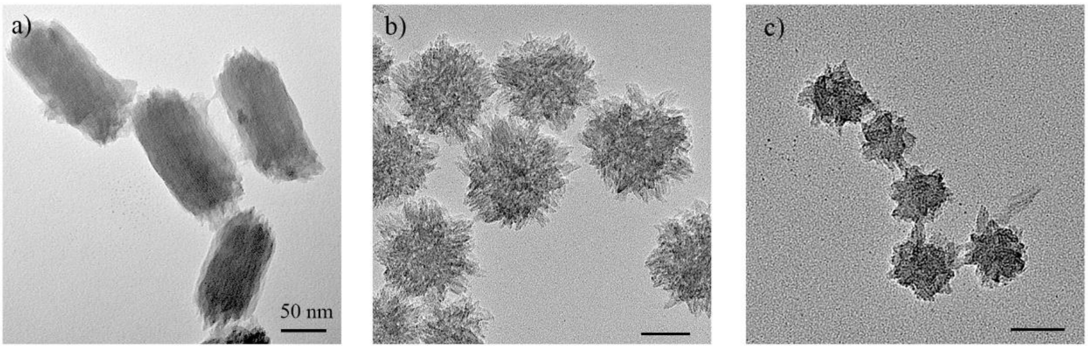

Supplementary Figure 6. MLNPs synthesised with a) 440 µL, b) 330 µL and c) 220 µL of NaOH solution (2 M). All the scale bars are 50 nm.

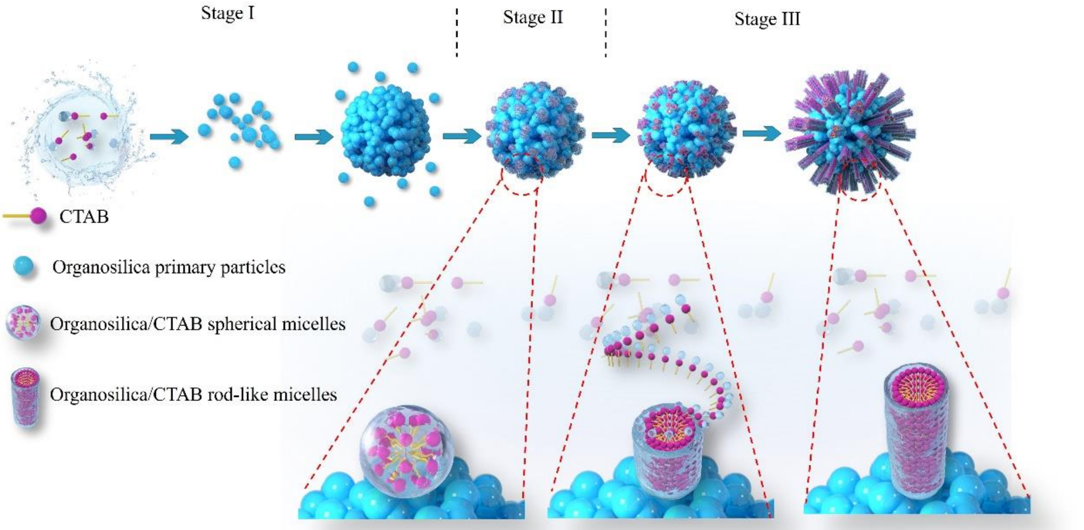

Supplementary Figure 7. Schematic illustration of the formation process. Stage I: Formation of organosilica primary particles and their aggregation and deposition into organosilica core. Stage II: Formation of spherical micelles as nucleation sites on the core particle surface. Stage III: Micelle phase transition-induced epitaxial growth of organosilica nanospikes.

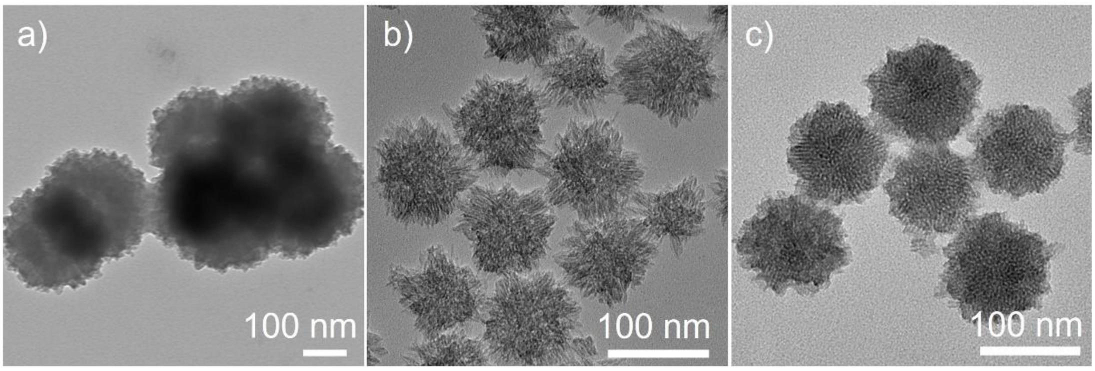

Supplementary Figure 8. The effect of stirring rate on formation of MLNPs. The stirring rate used for preparing the nanoparticles are a) 200 rpm, b) 500 rpm and c) 1000 rpm.

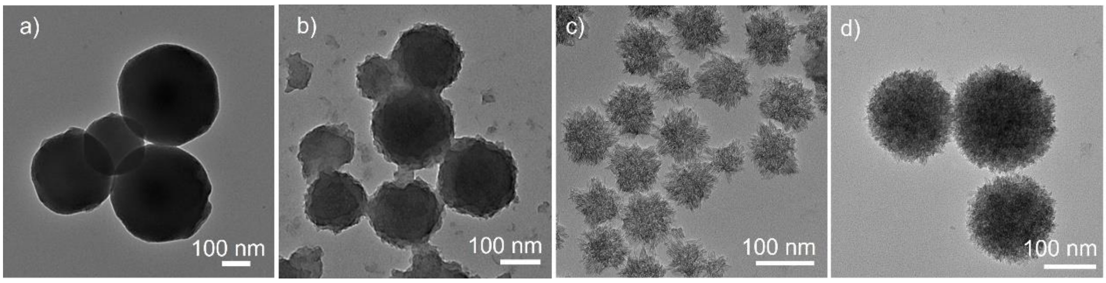

Supplementary Figure 9. The effect of temperature on formation of MLNPs. The temperature used for preparing the nanoparticles are a) 25 °C, b) 60 °C, c) 80 °C and d) 95 °C.

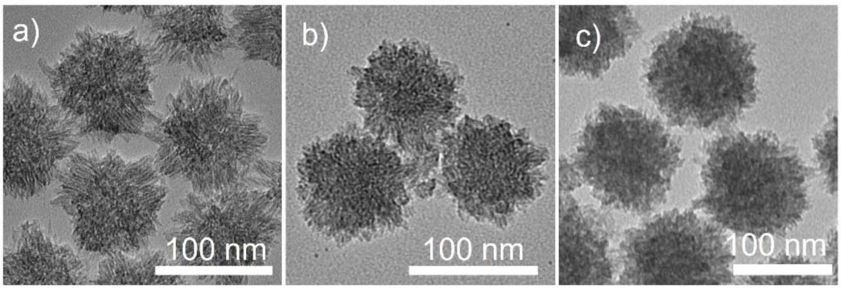

Supplementary Figure 10. The effect of the aging process on the formation of MLNPs. The aging time for the nanoparticles at a) 0 h, b) 4 h and c) 24 h.

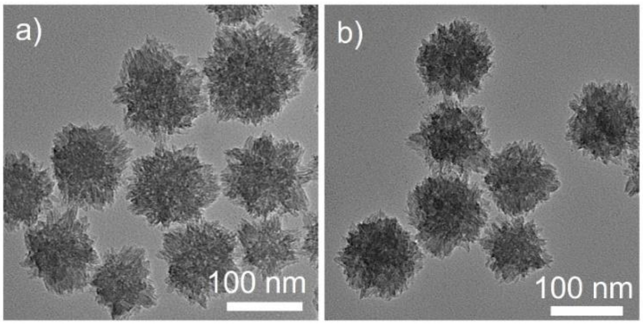

Supplementary Figure 11. The effect of the drying process on the formation of MLNPs. a) As-synthesized MLNPs without drying; b) As-synthesized MLNPs dried for 24 h at room temperature.

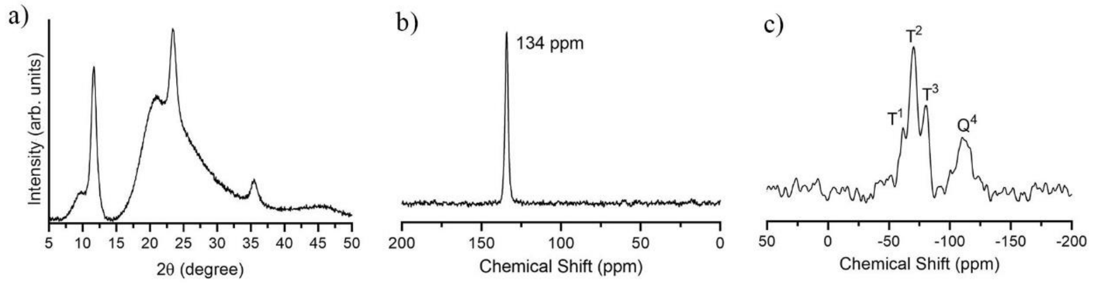

Supplementary Figure 12. Characterization of R/O-NPs. a) PXRD spectrum, solid state b) ^{13}C NMR and c) ^{29}Si NMR of R/O-NPs.

The powder X-Ray diffraction (PXRD) patterns displayed three sharp peaks at 11.6, 23.5 and 35.4° (2θ), corresponding to d = 7.6, 3.8 and 2.5 Å, respectively (Supplementary Figure 12a). The ^{13}C nuclear magnetic resonance (NMR) spectrum of R/O-NPs (Supplementary Figure 12b) shows a characteristic sharp peak at 134 ppm, corresponding to the C in −Si−C_6H_4−Si−. The ^{29}Si solid-state NMR spectrum exhibits peaks at -63, -71 and -81 ppm, which can be attributed to T^1 [C−Si(OSi)$(OX)_2$] (X = H or Et), T^2 [C−Si$(OSi)_2$(OX)] and T^3 [C−Si$(OSi)_3$] species, respectively (Supplementary Figure 12c). The presence of Q peak suggests a portion of cleaved Si-C bond during synthesis.

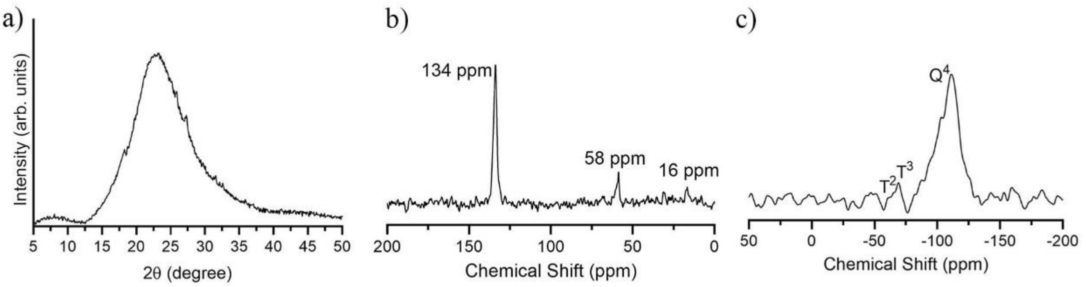

Supplementary Figure 13. Characterization of S/A-NPs. a) PXRD spectrum, solid state b) ^{13}C NMR and c) ^{29}Si NMR of S/A-NPs.

The powder XRD pattern displayed a broad peak at 23° (2θ), corresponding to amorphous silica (Supplementary Figure 13a). The ^{13}C nuclear magnetic resonance (NMR) spectrum of S/A-NPs shows a characteristic sharp peak at 134 ppm, corresponding to the C in $-Si-C_6H_4-Si-$ (Supplementary Figure 13b). The peaks at 58 ppm and 16 ppm can be attributed to unhydrolyzed ethoxy groups on BTEB. The ^{29}Si solid-state NMR spectrum exhibits peaks corresponding to T species and Q species (Supplementary Figure 13c).

It was observed that both MLNPs and R/O-NPs showed a small Q peak in the NMR spectra, and the intensities were comparable. The Q peak is due to the Si−C bond cleavage in the strong alkaline synthetic condition. In contrast, S/A-NPs showed a strong Q peak. This is because S/A-NPs have a silica framework (the main composition) and an organosilica surface, as they were prepared by calcination of MLNPs to remove organic contents and followed by post-modification of organosilica.

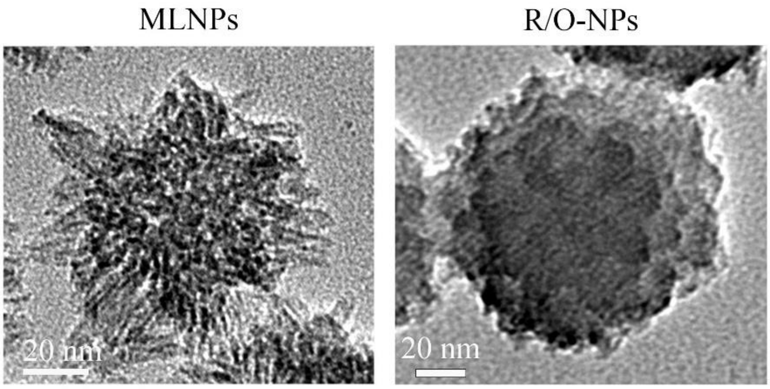

Supplementary Figure 14. TEM image of MLNPs and R/O-NPs at high magnification. R/O-NPs showed surface topography with certain degree of roughness, and no rod-like spike was observed on the surface. In contrast, MLNPs exhibited the spiky surface topography, on which rod-like spikes can be clearly observed.

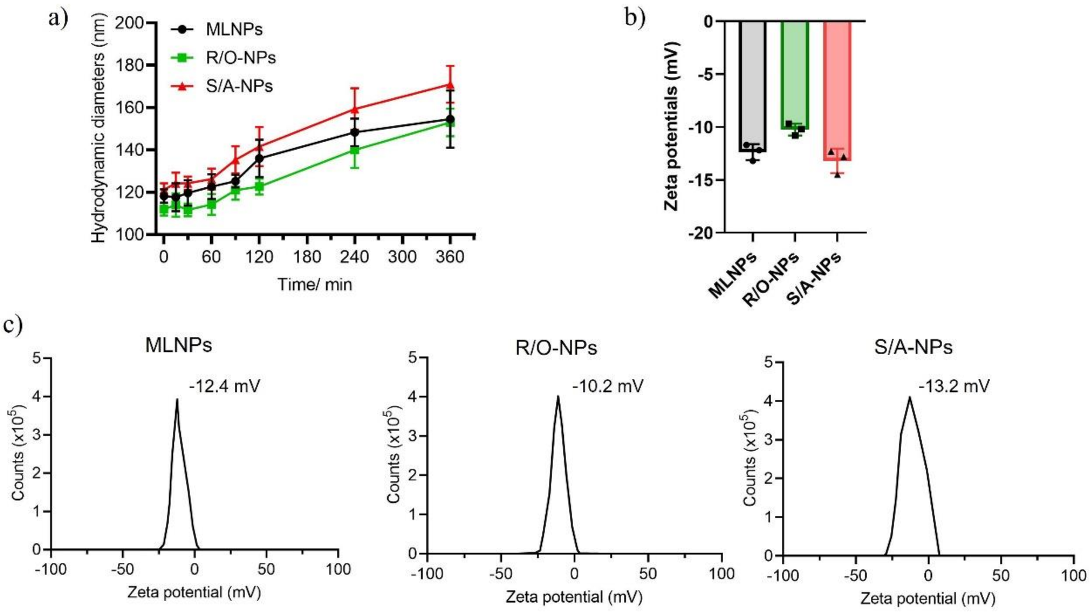

Supplementary Figure 15. a) Hydrodynamic diameters of nanoparticles after incubating in 6% bovine serum albumin solution for various time (n = 3 independent experiments). b) Zeta potentials of nanoparticles in water (n = 3 independent experiments). c) Zeta potential-intensity curves of nanoparticles (representative of three independent experiments).

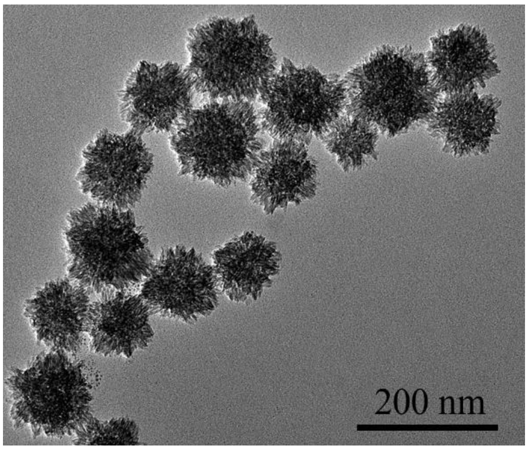

Supplementary Figure 16. The TEM image of MLNPs after immersing in PBS containing 5% human serum albumin at 37°C for 7 days.

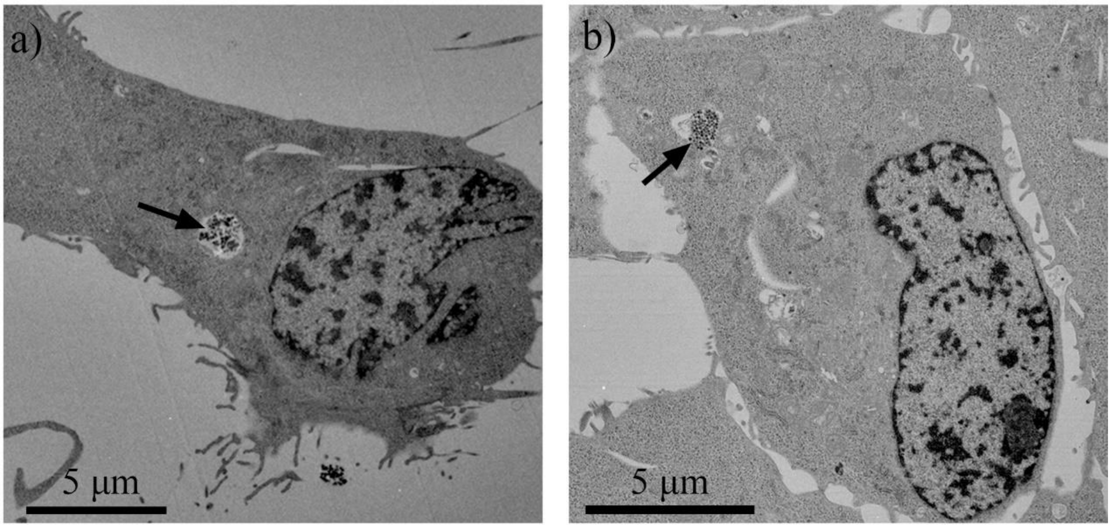

Supplementary Figure 17. Bio-TEM images of 4T1 cells after incubating with a) R/O-NPs and b) S/A-NPs for 6 h. Black arrows indicate the entrapment of nanoparticles in endo/lysosomes.

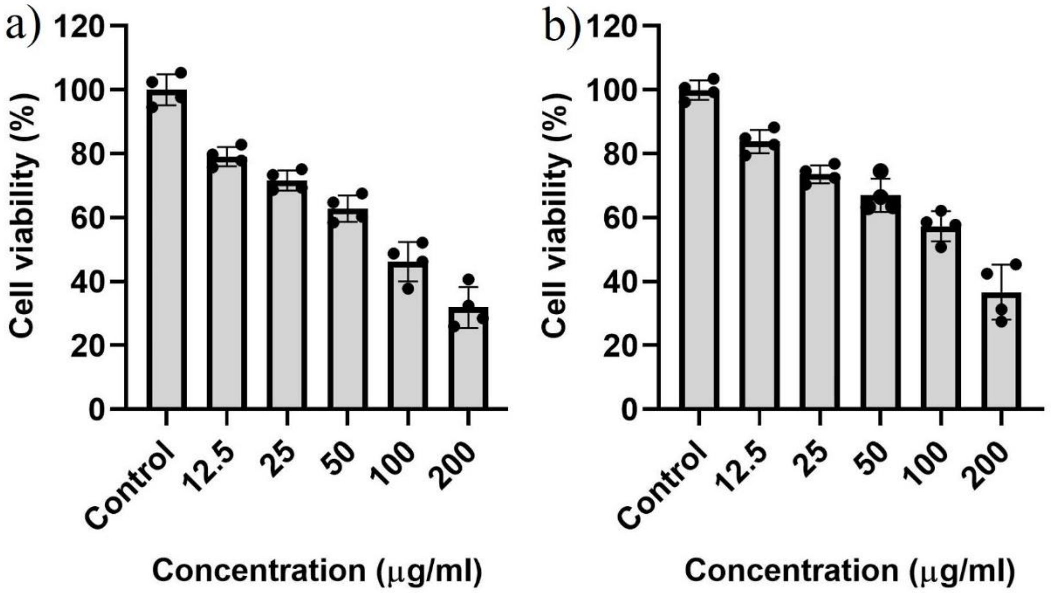

Supplementary Figure 18. The anti-proliferation activity of MLNPs at 48 h in a) DC2.4 and b) NIH-3T3 cell lines (n = 4 independent experiments).

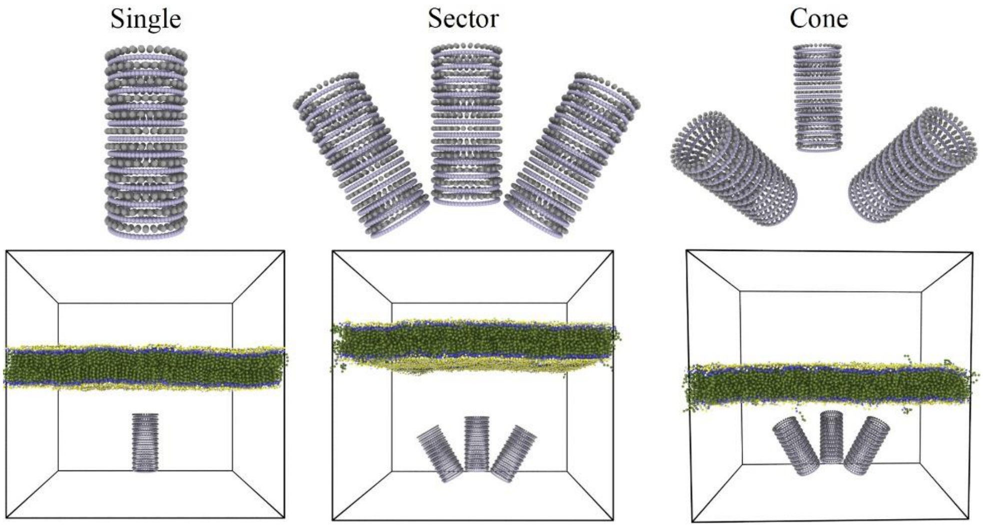

Supplementary Figure 19. Illustration of the simulation system, including single and triple nanospikes with sector- or cone-like arrangement and a bilayer membrane. Nanospikes were placed 12 nm blow the surface of the bilayer membrane system, and an external force of 12 kcal (mol^{-1} Å^{-1}) was applied to drag the nanospikes moving towards the membrane.

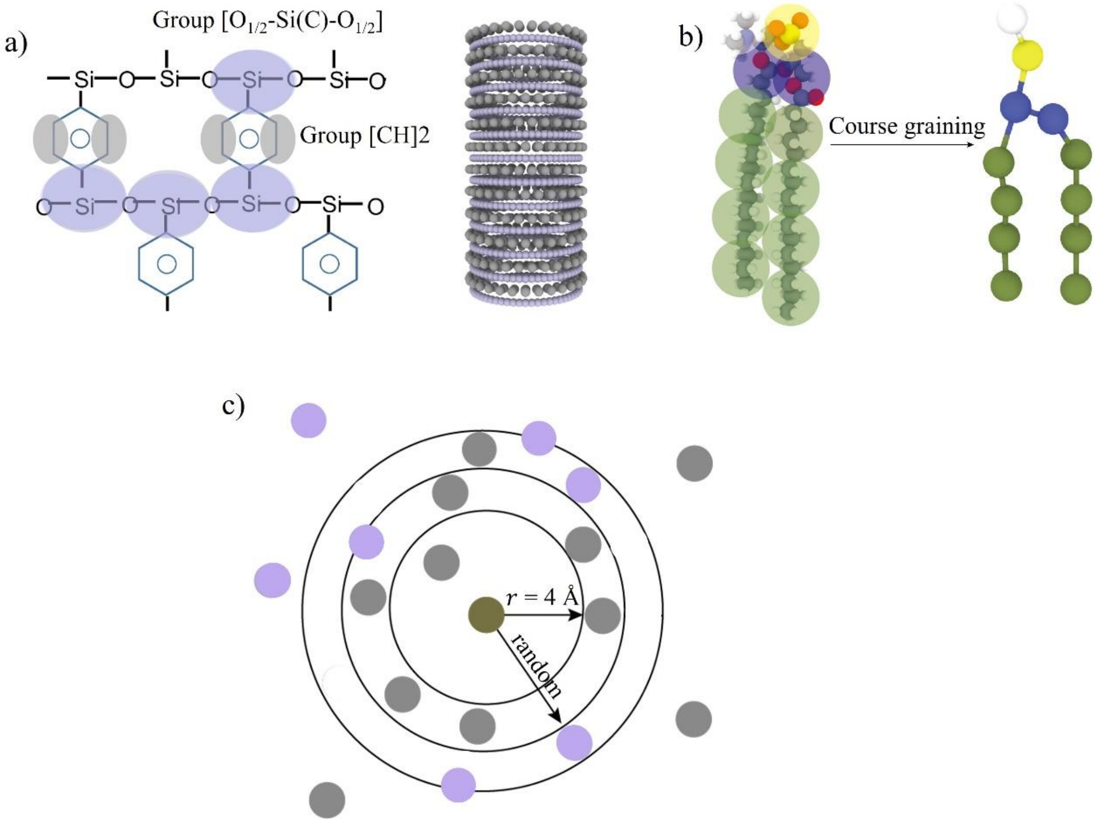

Supplementary Figure 20. Illustration of the individual components used in the

simulation. a) The coarse graining model of nanotube in Martini force field, where purple particles represent [$O_{1/2}$-Si(C)-$O_{1/2}$] group and grey particles represent [CH]$_2$ group. b) The coarse graining model of DPPC molecular in Martini force field. c) Scheme of the average distribution of [$O_{1/2}$-Si(C)-O $_{1/2}$] group (purple particles) and [CH]2 group (grey particles) around the phospholipid molecules (the central green particle).

In statistical mechanics, the radial distribution function (RDF) in a system of particles describes how density varies as a function of distance from a reference particle. In simplest terms it is a measure of the probability of finding a particle at a distance of r away from a given reference particle. The general algorithm involves determining how many particles are within a distance of r and $r + dr$ away from a particle. As illustrated in Supplementary Figure 20c, the central green particle is our reference particle (i.e., balls of the course-grained phospholipid molecules in Supplementary Figure 20b, and grey particles and purple particles are the balls of the course-grained [CH]$_2$ bond in benzene and [$O_{1/2}$-Si(C)-O $_{1/2}$] bond, respectively (Supplementary Figure 20a).

The information in Figure 4i is schematically illustrated in Supplementary Figure 20c. There is higher probability of grey particles distributed at r = 4 Å, while the distribution of purple particles is random and farther. The centralized distribution of grey particles at r = 4 Å suggests that phospholipid molecules tend to bind with benzene groups at a distance of 4 Å. In contrast, the random probability distribution suggests that the binding between phospholipid molecules and Si-O groups is unlikely to occur.

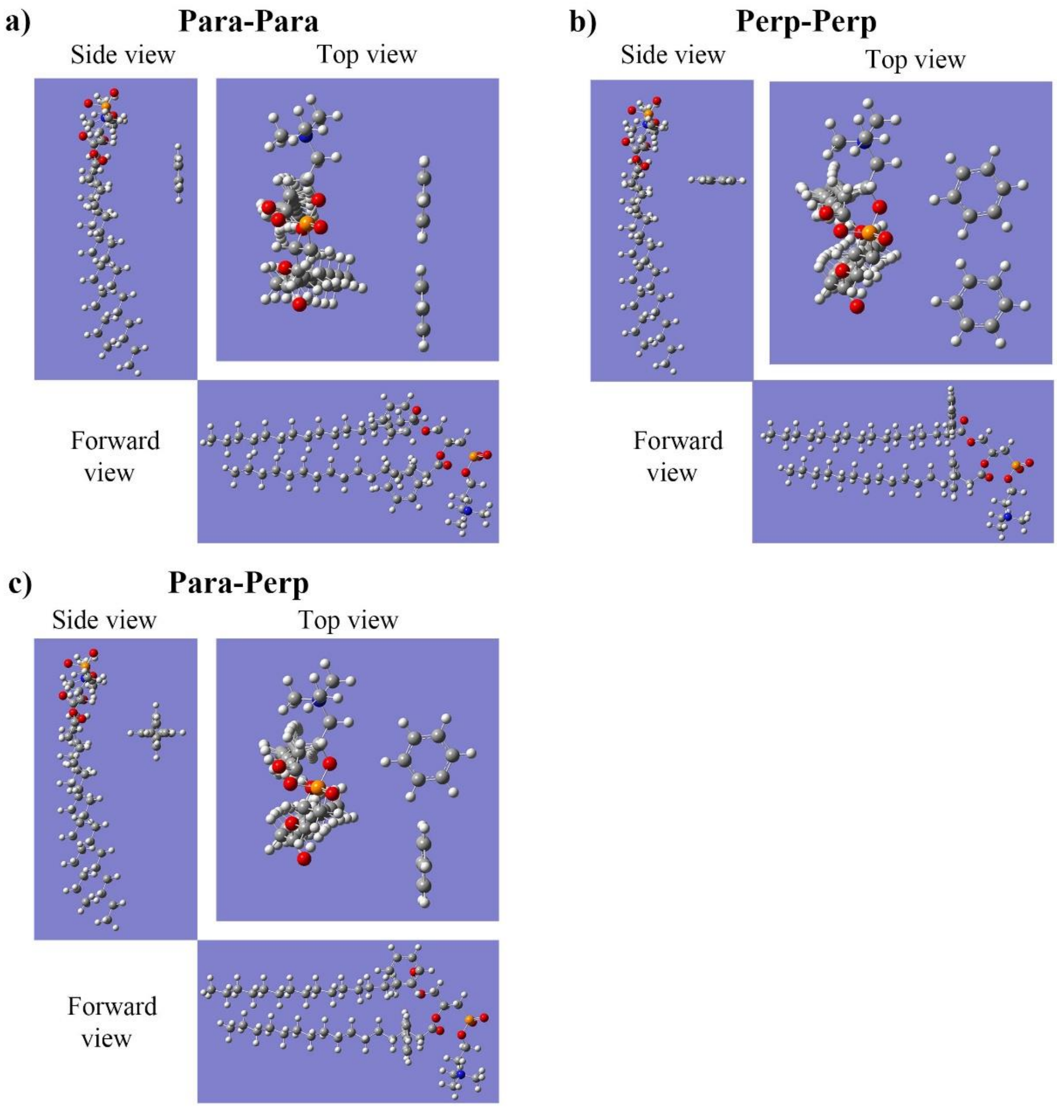

Supplementary Figure 21. Schematical illustration of the three models used in quantum chemistry calculation from side view, top view and forward views. a) Para-para: The planes of both benzene groups were parallel to the axial direction of the phospholipid molecule. b) Perp-Perp: The planes of both benzene groups were perpendicular to the axial direction of the phospholipid molecule. c) Para-Perp: The plane of one benzene group was parallel while the other was perpendicular to the axial direction of the phospholipid molecule.

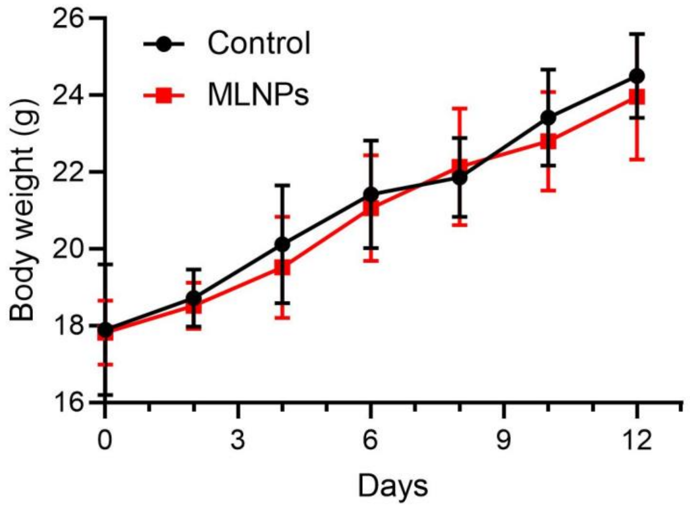

Supplementary Figure 22. The body weight of mice received with three doses of intratumoral injections of PBS (control) or MLNPs (30 mg/kg for each dose) (n = 5 biologically independent animals).

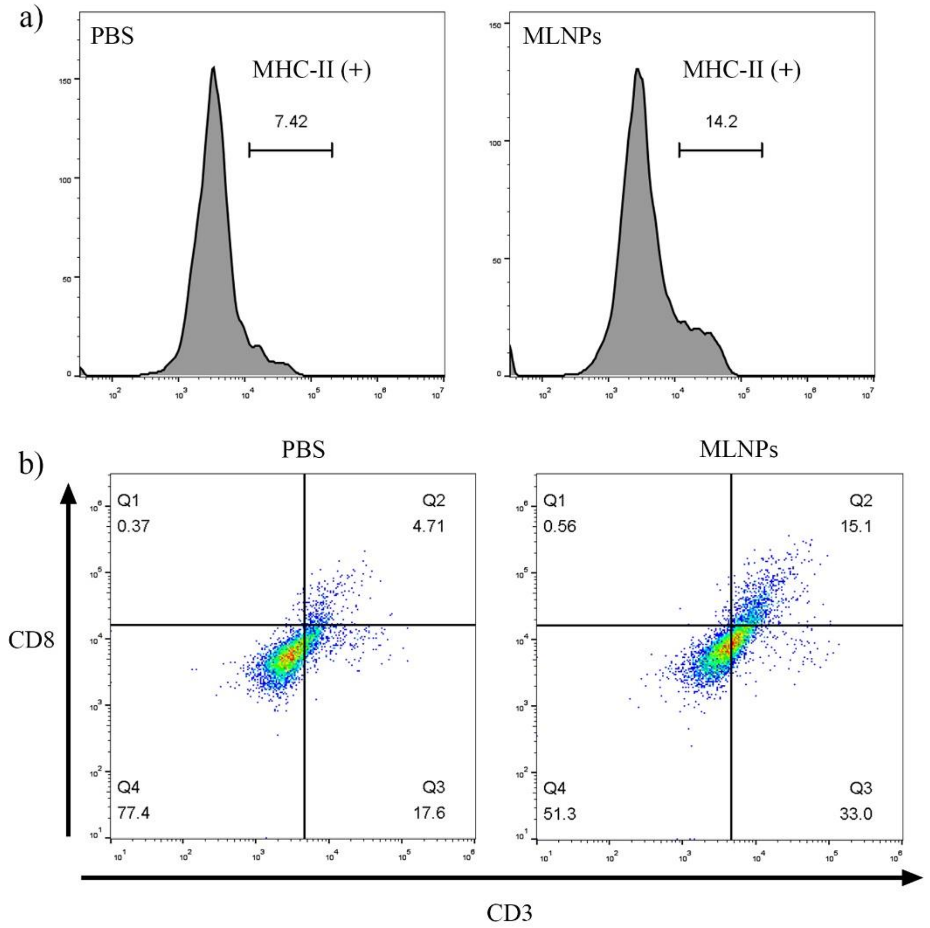

Supplementary Figure 23. a) FACS plots of the MHC-II$^+$ DC population and b)

CD3^{+}CD8^{+} T cells population in the tumours of mice injected with PBS or MLNPs.

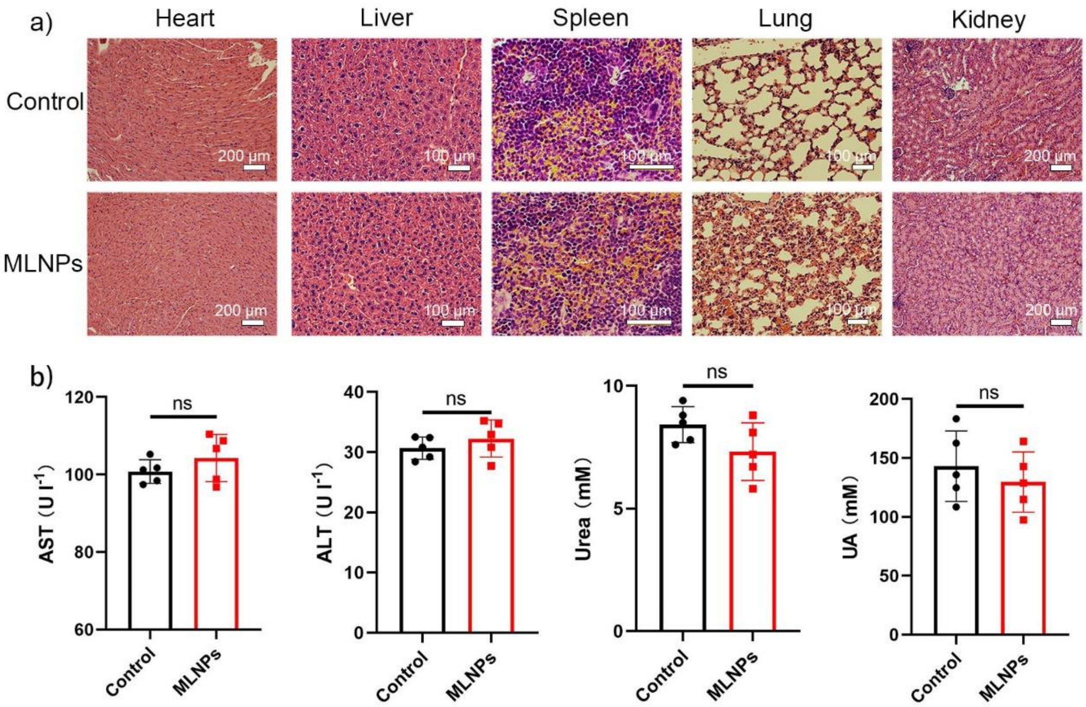

Supplementary Figure 24. a) H&E staining of heart, liver, spleen, lung, and kidney 7 days after three injections of PBS (control) or MLNPs (50 mg kg^{-1}). b) The concentrations of aspartate transferase (AST), alanine transaminase (ALT), urea and uric acid (UA) in the serum 7 days after three injections of PBS (control) or MLNPs at a dose of 50 mg kg^{-1} to evaluate their hepatotoxicity and nephrotoxicity (n=5 biologically independent animals). Statistical significance was determined by unpaired t-test. ns = non-significant.

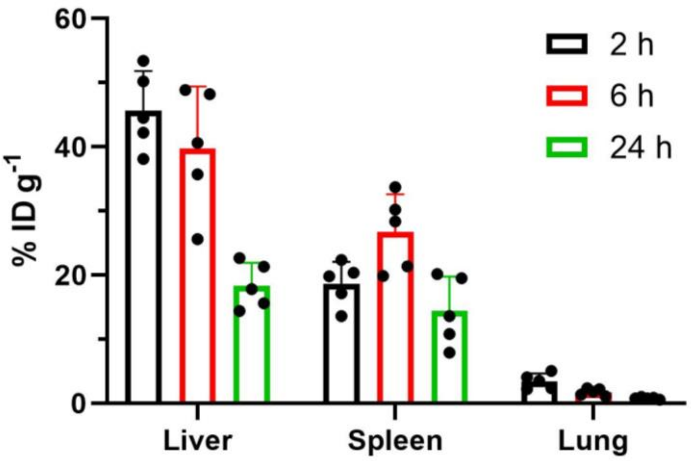

Supplementary Figure 25. Biodistribution of MLNPs in the RES organs (liver, spleen and lung) at 2, 6 and 24 h post intravenous injection (n = 5 biologically independent animals). The dose of MLNPs was 10 mg kg^{-1}.

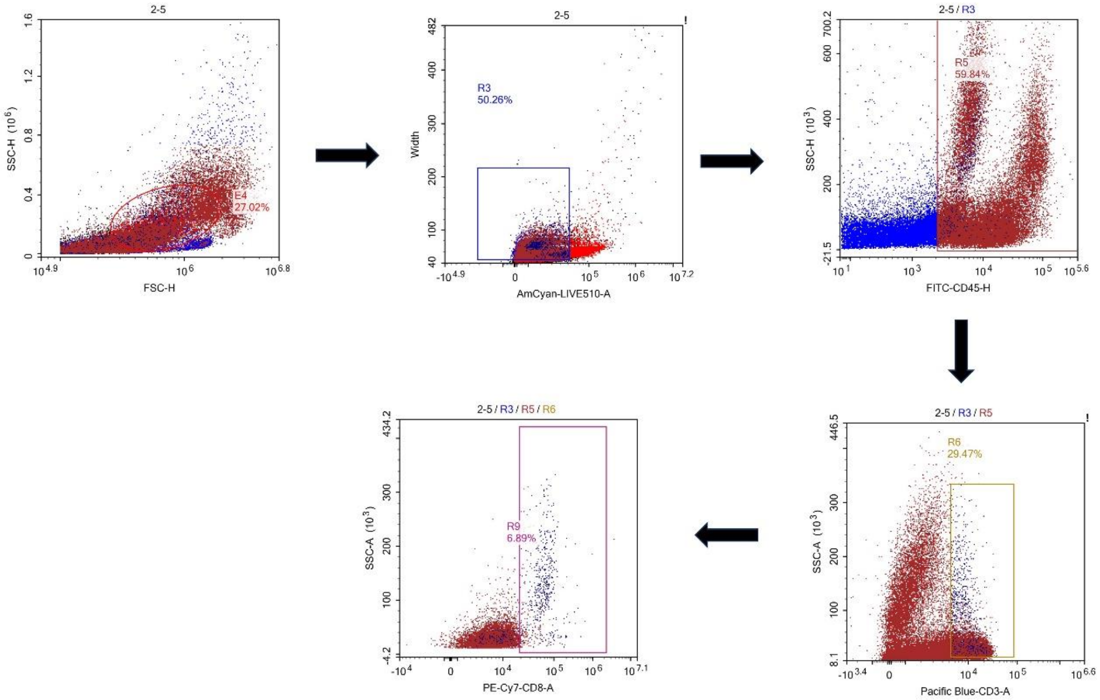

Supplementary Figure 26. Representative scatter plots and gating information derived from analysis of CD8+ T cells in tumour.
