# Supplementary material for: Mesoporous nanoperforators as membranolytic agents via nano- and molecular-scale multi-patterning
Source: Nat Commun. 2024 Feb 29;15:1891. doi: 10.1038/s41467-024-46189-9 (PMC10904871; doi:10.1038/s41467-024-46189-9)
Supplement: Supplementary file 2 — Reporting Summary [file 41467_2024_46189_MOESM2_ESM.pdf]

nature portfolio

Corresponding author(s): Yannan Yang, Min Zhang, Xiao He and Chengzhong Yu

Last updated by author(s): Jan 30, 2024

Reporting Summary

Nature Portfolio wishes to improve the reproducibility of the work that we publish. This form provides structure for consistency and transparency in reporting. For further information on Nature Portfolio policies, see our Editorial Policies and the Editorial Policy Checklist.

Statistics

For all statistical analyses, confirm that the following items are present in the figure legend, table legend, main text, or Methods section.

n/a	Confirmed	
☐	☒	The exact sample size (*n*) for each experimental group/condition, given as a discrete number and unit of measurement
☐	☒	A statement on whether measurements were taken from distinct samples or whether the same sample was measured repeatedly
☐	☒	The statistical test(s) used AND whether they are one- or two-sided<br>*Only common tests should be described solely by name; describe more complex techniques in the Methods section.*
☒	☐	A description of all covariates tested
☒	☐	A description of any assumptions or corrections, such as tests of normality and adjustment for multiple comparisons
☐	☒	A full description of the statistical parameters including central tendency (e.g. means) or other basic estimates (e.g. regression coefficient) AND variation (e.g. standard deviation) or associated estimates of uncertainty (e.g. confidence intervals)
☐	☒	For null hypothesis testing, the test statistic (e.g. *F*, *t*, *r*) with confidence intervals, effect sizes, degrees of freedom and *P* value noted<br>*Give P values as exact values whenever suitable.*
☒	☐	For Bayesian analysis, information on the choice of priors and Markov chain Monte Carlo settings
☒	☐	For hierarchical and complex designs, identification of the appropriate level for tests and full reporting of outcomes
☒	☐	Estimates of effect sizes (e.g. Cohen's *d*, Pearson's *r*), indicating how they were calculated

Our web collection on statistics for biologists contains articles on many of the points above.

Software and code

Policy information about availability of computer code

Data collection	BD FACS Diva software v8.0.1.1 (flow cytometry)<br>ZEISS ZEN2 software (confocal)<br>Digital pathology scanner software (histopathology)<br>Gaussian 16 program with the wB97XD/6-311+G*
Data analysis	FlowJo software V10.0.7 (flow cytometry)<br>ZENISS ZEN2 software (Confocal)<br>Graphpad Prism v8.0 (data plotting and statistical analysis)<br>Gaussian 16 program with the wB97XD/6-311+G*

For manuscripts utilizing custom algorithms or software that are central to the research but not yet described in published literature, software must be made available to editors and reviewers. We strongly encourage code deposition in a community repository (e.g. GitHub). See the Nature Portfolio guidelines for submitting code & software for further information.

Data

Policy information about availability of data

All manuscripts must include a data availability statement. This statement should provide the following information, where applicable:

- Accession codes, unique identifiers, or web links for publicly available datasets
- A description of any restrictions on data availability
- For clinical datasets or third party data, please ensure that the statement adheres to our policy

The data that support the findings of this study are available within the article and its Supplementary Information files. Data generated in this study are provided in the Source Data file.

Research involving human participants, their data, or biological material

Policy information about studies with human participants or human data. See also policy information about sex, gender (identity/presentation), and sexual orientation and race, ethnicity and racism.

Reporting on sex and gender	N/A
Reporting on race, ethnicity, or other socially relevant groupings	N/A
Population characteristics	N/A
Recruitment	N/A
Ethics oversight	N/A

Note that full information on the approval of the study protocol must also be provided in the manuscript.

Field-specific reporting

Please select the one below that is the best fit for your research. If you are not sure, read the appropriate sections before making your selection.

☒ Life sciences ☐ Behavioural & social sciences ☐ Ecological, evolutionary & environmental sciences

For a reference copy of the document with all sections, see nature.com/documents/nr-reporting-summary-flat.pdf

Life sciences study design

All studies must disclose on these points even when the disclosure is negative.

Sample size	Sample sizes were determined based on estimates from pilot experiments, as statistical methods were not used for sample size calculation. For in vitro experiments, at least triplicates were included to enable statistical calculations. For in vivo studies, a sample size of 4-5 animals per treatment group was deemed sufficient to reliably detect statistically significant differences. Detailed sample size for each experiment are provided in the methods section or figure caption.
Data exclusions	No data were excluded from the analysis.
Replication	All in vitro cell studies included 3-4 biologically independent samples or independent experiments. All reported attempts at replication were successful. For in vivo studies, each treatment groups consist of 4-5 mice. In vivo experiments consist of a least one preliminary experiment and a formal treatment experiment.
Randomization	All samples, cells and mice were allocated randomly in experiment groups.
Blinding	Investigators were not blinded as they designed the experiments and also performed the investigations. Therefore, the investigators maintained awareness of the experimental conditions throughout the data acquisition and analysis process. However, the investigators were unbiased in both outcome assessment and conclusion drawing. Critical experiments were analyzed by at least two independent investigators for unbiased conclusion.

Reporting for specific materials, systems and methods

We require information from authors about some types of materials, experimental systems and methods used in many studies. Here, indicate whether each material, system or method listed is relevant to your study. If you are not sure if a list item applies to your research, read the appropriate section before selecting a response.

Materials & experimental systems

n/a	Involved in the study
	☒ Antibodies
	☒ Eukaryotic cell lines
☒	☐ Palaeontology and archaeology
	☒ Animals and other organisms
☒	☐ Clinical data
☒	☐ Dual use research of concern
☒	☐ Plants

Methods

n/a	Involved in the study
☒	☐ ChIP-seq
	☒ Flow cytometry
☒	☐ MRI-based neuroimaging

Antibodies

Antibodies used

anti-mouse CD16/32, Catalog#553142, RRID: AB_394656, Clone 2.4G2 (RUO), BD Biosciences (1:160 dilution)
anti-mouse CD45-FITC, Catalog#553080, RRID: AB_394609, Clone 30-F11 (RUO), BD Biosciences (1:200 dilution)
anti-mouse CD3-BV421, Catalog#562600, RRID: AB_11153670, Clone 145-2C11 (RUO), BD Biosciences (1:200 dilution)
anti-mouse CD8-PE-CY7, Catalog#552877, RRID: AB_394506, Clone 53-6.7 (RUO), BD Biosciences (1:160 dilution)
anti-mouse F4/80-BV421, Catalog#565411, RRID: AB_2734779, Clone T45-2342 (RUO), BD Biosciences (1:160 dilution)
anti-mouse IA-IE (MHC-II)-BV786, Catalog#743875, RRID: AB_2741826, Clone 2G9 (RUO), BD Biosciences (1:160 dilution)
anti-mouse CD11C-PE-CY7, Catalog#561022, RRID: AB_647251, Clone HL3 (RUO), BD Biosciences (1:200 dilution)

Validation

Validation details of the primary antibodies are available on the manufacturers' websites:
Anti-mouse CD16/32
https://www.bdbiosciences.com/en-au/products/reagents/flow-cytometry-reagents/research-reagents/single-color-antibodies-ruo/purified-rat-anti-mouse-cd16-cd32-mouse-bd-fc-block.553142
Anti-mouse CD45-FITC
https://www.bdbiosciences.com/en-au/products/reagents/flow-cytometry-reagents/research-reagents/single-color-antibodies-ruo/fitc-rat-anti-mouse-cd45.553080
Anti-mouse CD3-BV421
https://www.bdbiosciences.com/en-au/products/reagents/flow-cytometry-reagents/research-reagents/single-color-antibodies-ruo/bv421-hamster-anti-mouse-cd3e.562600
Anti-mouse CD8-PE-CY7
https://www.bdbiosciences.com/zh-cn/products/reagents/flow-cytometry-reagents/research-reagents/single-color-antibodies-ruo/pe-cy-7-rat-anti-mouse-cd8a.552877
Anti-mouse F4/80-BV421
https://www.bdbiosciences.com/en-au/products/reagents/flow-cytometry-reagents/research-reagents/single-color-antibodies-ruo/bv421-rat-anti-mouse-f4-80.565411
Anti-mouse IA-IE (MHC-II)-BV786
https://www.bdbiosciences.com/en-au/products/reagents/flow-cytometry-reagents/research-reagents/single-color-antibodies-ruo/bv786-rat-anti-mouse-i-a-i-e.743875
Anti-mouse CD11C-PE-CY7
https://www.bdbiosciences.com/en-au/products/reagents/flow-cytometry-reagents/research-reagents/single-color-antibodies-ruo/pe-cy-7-hamster-anti-mouse-cd11c.561022

Eukaryotic cell lines

Policy information about cell lines and Sex and Gender in Research

Cell line source(s)

The mouse cell lines, including 4T1 and NIH/3T3 were obtained from the American Type Culture Collection (ATCC). DC2.4 cell line was obtained from YoBiBiotech Co., Ltd.

Authentication

Cell lines were authenticated by identification of short tandem repeat (STR) markers

Mycoplasma contamination

Cell lines were tested negative for mycoplasma contamination.

Commonly misidentified lines
(See ICLAC register)

No commonly misidentified cell lines were used.

Animals and other research organisms

Policy information about studies involving animals; ARRIVE guidelines recommended for reporting animal research, and Sex and Gender in Research

Laboratory animals

Female Balb/c mice (6-8 weeks) were purchased from Shanghai Lingchang Biotechnology Co. Ltd.. Animals were bred and housed in a standard barrier animal facility at Fudan University with the light cycle of 14:10, ambient temperature at 22 °C, and relative humidity range between 30-70%. Experimental animals were randomly assigned to into different treatment. Experimental and control animals were co-housed. All animal-related experiments were performed in full compliance with animal protocols approved by Fudan

	University Institutional Animal Care and Use Committee.
Wild animals	No wild animals were included.
Reporting on sex	Female mice were used in this study.
Field-collected samples	No field-collected samples were used.
Ethics oversight	This research complies with all relevant ethical regulations. Experiments were performed in agreement with the Animal Ethics Committee of Fudan University.

Note that full information on the approval of the study protocol must also be provided in the manuscript.

Flow Cytometry

Plots

Confirm that:

- [x] The axis labels state the marker and fluorochrome used (e.g. CD4-FITC).
- [x] The axis scales are clearly visible. Include numbers along axes only for bottom left plot of group (a 'group' is an analysis of identical markers).
- [x] All plots are contour plots with outliers or pseudocolor plots.
- [x] A numerical value for number of cells or percentage (with statistics) is provided.

Methodology

Sample preparation	At the end of tumour treatment, mice were sacrificed, and the tumour tissues were collected. The tumour tissues were dissociated into single cell suspension by enzymic digestion in RPMI160 containing type IV collagenase (1mg/ml), deoxyribonuclease (100 ug/ml) and hyaluronidase (100 ug/ml) at 37 degree for 25 min. Digested cells were passed through a 40 um nylon mesh and collected by centrifugation at 300 rpm for 10 mins, followed by red blood cell lysis. 100 ul of cell suspension were used for flow cytometry analysis.
Instrument	BD FACSymphony A5, BD Fortessa LSRII
Software	BD FACS Diva Software, FlowJo software
Cell population abundance	No sorting was performed.
Gating strategy	FSC-A vs SSC-A was used to exclude cell debrids, followed by FSC-A vs FSC-H to determine singlets. Live cells were then gated based on viability dye staining. Dendritic cells were gated as CD11c+ F4/80– CD45+, and CD8+ T cells were gated as CD45+ CD3+ CD8+.

- [x] Tick this box to confirm that a figure exemplifying the gating strategy is provided in the Supplementary Information.
